# Resistance Status of the Colorado Potato Beetle, *Leptinotarsa decemlineata,* to Endosulfan in East Azarbaijan and Ardabil Provinces of Iran

**DOI:** 10.1673/031.007.3101

**Published:** 2007-05-14

**Authors:** M. Mohammadi Sharif, M. J. Hejazi, A. Mohammadi, M. R. Rashidi

**Affiliations:** ^1^ Department of Plant Protection, Faculty of Agriculture, University of Tabriz, Tabriz, Iran; ^2^ Department of Plant Breeding, Faculty of Agriculture, University of Tabriz, Tabriz, Iran; ^3^ Drug Applied Research Center, Tabriz University of Medical Sciences, Tabriz, Iran

**Keywords:** synergism, S, S, S-tributylphosphorotrithioate, piperonyl butoxide, resistance mechanism

## Abstract

Three Colorado potato beetle, *Leptinotarsa decemlineata*, populations, from Ardabil, Bostanabaad and Ajabshir, were collected from potato fields in East Azarbaijan and Ardabil provinces in Iran and assayed for resistance to endosulfan. Possible resistance mechanisms were investigated using synergism studies and biochemical assays. Laboratory tests showed that the Bostanabaad strain was 220 and 109 times resistant compared with the susceptible strain in 2003 and 2004, respectively. The resistance ratios for the Ajabshir and Ardabil strains were 19 and 18, respectively. Since considerably more resistance was observed in the Bostanabaad strain compared with the other two, further investigation of the origin of resistance was done on this strain. Two insecticide synergists, piperonyl butoxide and S,S,S-tributylphosphorotrithioate, reduced resistance 2.3 and 3.5 times, respectively. These small degrees of synergism suggest that metabolism is not the source of the considerable difference in susceptibility between the two strains. This was supported by the results obtained from the biochemical assays that showed that glutathione S-transferase activity in the Bostanabaad strain did not significantly differ from the susceptible strain. These results suggest that target site insensitivity may be involved.

## Introduction

The Colorado potato beetle, *Leptinotarsa decemlineata* (Say), is a major pest of potatoes in most potato growing regions of the world, including Iran. In the absence of control practices, *L. decemlineata* populations can completely defoliate potato plants. In general, potato plants are sensitive to beetle defoliation up to 60 days after planting (Zehnder and Evanylo 1989). However, most of the damage is caused at the beginning of blooming, which coincides with tuber formation and filling (Hare 1990). This insect was a quarantine pest in Iran, but in 1984 was reported from Ardabil, the major potato production region of Iran (Nouri Ganbalani 1986).

*L. decemlineata* control mainly involves the use of insecticides. Development of resistance to organochlorine insecticides by *L. decemlineata* occurred quickly (McDonald 1976; Harris and Svec 1976; Harris and Svec 1981) and is widespread. Resistance to organophosphorous, carbamate and pyrethroid insecticides has been documented in many locations (Harris and Svec 1981; Heim et al. 1990; French et al. 1992; Pap et al. 1997). This insect has a high potential for the development of resistance against different insecticidal chemicals including recently registered products (Zhao et al. 2000; Cutler et al. 2005). Three main reasons for this high potential are its high capability for increasing its population in a short period of time (Senanayake et al. 2000), the selection of resistant individuals due to a higher chance of exposure of all active stages to insecticides (Follett et al. 1993), and cross resistance to several insecticides as a result of a single resistance mechanism or the co-existence of several resistance mechanisms leading to multiple resistance to several insecticides (French et al. 1992). Besides these, other factors such as the absence of crop rotation in some regions, and the occurrence of local populations of *L. decemlineata*, may lead to homozygocity for insecticide resistance.

Since the development of resistance can affect the efficiency of available insecticides for *L. decemlineata* control, understanding the resistance status of *L. decemlineata* populations would be useful (French et al. 1992). A successful resistance management strategy involves a thorough knowledge of resistance mechanisms (Ioannidis et al. 1992).

Application of insecticide synergists is one of the easiest and fastest procedures to obtain preliminary information about potential mechanisms of resistance (Scott 1990). Piperonyl butoxide inhibits cytochrome P450 microsomal monooxygenases, and S,S,S-tributylphosphorotrithioate (DEF) inhibits esterases and glutathione S-transferase. Since endosulfan does not have an ester bond, in such synergism studies, DEF acts as glutathione S-transferase inhibitor (Rufingier et al. 1999).

Endosulfan is both an effective insecticide against *L. decemlineata*, and is economical, so it has been well accepted and used for several years by potato growers in many potato growing regions of northwestern Iran. Hence, determining the existence and possible causal mechanisms of resistance to this insecticide is of prime importance. To achieve this goal, endosulfan was tested on 4th instar larvae of the *L. decemlineata* populations and possible mechanisms of resistance were investigated using synergism studies and biochemical assays.

## Materials and Methods

### Insect strains and rearing conditions

During 2003 and 2004, populations of Colorado potato beetle were collected from potato fields of Ardabil county in the province of Ardabil, and Bostanabaad and Ajabshir counties in East Azarbaijan province. In Bostanabaad the field sampled in 2004 was different from the sampled field in 2003. A susceptible strain was supplied by M. S. Goettel, Agriculture and Agri-Food Canada Research Center, Lethbridge. Colonies of these insects were reared in greenhouse at 26 ± 2° C, 50 ± 5% RH and 16:8 (L:D) photoperiod on unsprayed potato foliage, and were monitored for insecticide resistance.

### Chemicals

Technical grade endosulfan (96% purity) was supplied by Bayer Cropscience (bayercropscience.com). S,S,S-tributylphosphorotrithioate (DEF) and piperonyl butoxide were purchased from Chem Service (chemservice.com) and Fluka Chemie (sigmaaldrich.com), respectively. 1-chloro-2, 4-dinitrobenzene (CDNB) and reduced glutathione were purchased from Merck (merck.de), copper sulfate and bicinchoninic acid from Sigma-Aldrich Chemie (sigmaaldrich.com) and bovin serume albumin and ethylene diamine tetraacetic acid were purchased from Carl Roth (carl-roth.de).

**Table 1.**
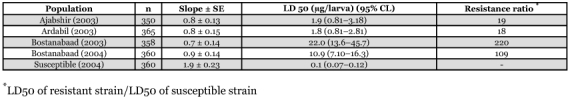
Resistance status of *Leptinotarsa decemlineata* populations

### Bioassays

Bioassays were conducted using 24–48 hour old 4^th^ instar larvae. To obtain uniform larvae for use in bioassays, up to 24 hour old larvae were fed for 24 hours under rearing conditions. The insecticide was dissolved in acetone and 0.5 (in 2003) or 1.0 µl (in 2004) aliquots were deposited on the mesonotum. The control larvae were treated with 0.5 or 1.0 µl acetone. The ranges of endosulfan doses for susceptible and resistant strains were 0.05–0.5 and 1–80 µg/larva, respectively. Technical difficulties made it necessary to change the droplet size to 1 µl in 2004, which is likely to have increased the area of the cuticle surface treated.

The treated larvae were placed in 13 × 6.5 × 4 cm transparent plastic containers and supplied with untreated potato foliage. The treated insects were kept at 26 ± 1° C and 16:8 (L:D) photoperiod and mortality was recorded 24 h after treatment. The affected larvae were considered alive if they could move their legs and body after touching one leg with a needle. Five levels of the insecticide were used in all treatments and each treatment consisted of 4–5 replicates. The probit option of SPSS was used for analyzing dose-mortality data (SPSS 1999).

### Synergism studies

The synergists used in this study were DEF and piperonyl butoxide. These chemicals were dissolved in acetone and applied topically one hour prior to treatment with endosulfan using the same procedure described above. Each treated larva received 5 µg DEF or 10 µg piperonyl butoxide. These were the highest doses of the synergists that showed no mortality on the susceptible strain. The control larvae were treated with piperonyl butoxide or DEF. The insecticide doses and the procedure used for data analysis were similar to those described above. Each treatment was replicated four times.

**Table 2.**
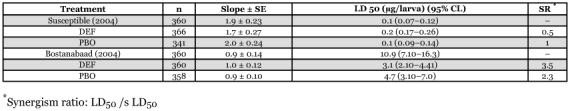
Synergism of endosulfan with DEF and PBO in resistant and susceptible populations of *Leptinotarsa decemlineata*

### Biochemical assays

Glutathione S-transferase activity was measured using CDNB (Habig et al. 1974). Abdomens of fourth instar larvae were homogenized in phosphate buffer (100 mM, pH 6.5) containing 5 mM reduced glutathione. The homogenate was centrifuged at 11000 g and 4°^c^, and the supernatant was used as the enzyme source. In a final volume of 3 ml, the reaction mixture consisted of 50 µl supernatant, 50 µl 1 mM CDNB in methanol and 2.9 ml of the buffer. After adding CDNB, the reaction was assayed spectrophotometrically (Shimadzo, UV-2550) at 340 nm for 3 min and the activity was calculated using the extinction coefficient of 9.6 mM^-1^cm^-1^. A blank consisted of 50 µl CDNB and 2.95 ml of the phosphate buffer; each treatment was replicated four times. To determine Michaelis-Menten parameters, the initial velocity of reactions resulting from six different concentrations of CDNB (0.05-1 mM) with the enzyme fraction were measured separately in both susceptible and resistant strains. The Km and Vmax values were obtained from Lineweaver-Burk plot. These treatments were replicated three times.

Protein content was measured with bicinchoninic acid procedure (Smith et al. 1985) using bovine serum albumin as the standard.

**Table 3.**
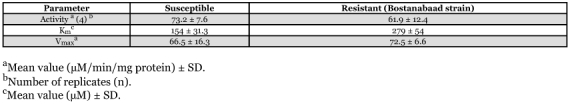
Activity and kinetic parameters of GSH-transferase of susceptible and resistant strains of *Leptinotarsa decemlineata*

## Results

Compared with the susceptible strain, the Bostanabaad strain was 220 and 109 times resistant to endosulfan in 2003 and 2004, respectively. The resistance ratios for Ajabshir and Ardabil strains were 19 and 18, respectively (Table 1). As shown in Table 2, piperonyl butoxide and DEF did not synergize endosulfan in the susceptible strain, but enhanced the toxicity of endosulfan in the resistant Bostanabaad strain, piperonyl butoxide and DEF reduced resistance 2.3 and 3.5 times, respectively. Glutathione S-transferase activity of susceptible and resistant *L. decemlineata* strains was measured using in *vitro* CDNB (Table 3). No statistical difference (*P* > 0.05) was observed between the two strains. There was also no difference (*P* > 0.05) in Vmax for glutathione S-transferase activity toward CDNB between the susceptible and resistant strains. Affinity of glutathione S-transferase for CDNB, as addressed by higher Km in resistant vs. susceptible strains, was significantly (*P* < 0.05) lower in the resistant strain.

## Discussion

Monooxygenase and glutathione S-transferase based metabolism have been shown to be associated with insecticide resistance in *L. decemlineata*. Argentine et al. 1989 demonstrated that these enzyme systems were involved in resistance to azinphosmethyl and permethrin. Other investigations have also shown the importance of detoxifying mechanisms in *L. decemlineata* resistant strains (Argentine et al. 1992; Ahammad-Sahib et al. 1994; Zhao et al. 2000).

Our results indicate that oxidases and glutathione S-transferases may be involved in endosulfan metabolism in the resistant Bostanabaad population, but may not be solely responsible for endosulfan resistance in this strain because the treatment with the synergists could not cope with the resistance problem completely. This suggests that other mechanisms may be involved as well. Lin et al. 1993 also did not find metabolic detoxification as an important mechanism for dieldrin resistance in *Tribolium castaneum*.

Although glutathione S-transferase is a major detoxifying enzyme system in insecticide detoxification (Hemingway 2000; Kostaropoulos et al. 2001), our results from the biochemical assays are in agreement with the synergism data and confirm only a minor effect of glutathione S-transferase in detoxifying endosulfan in the resistant strain of the *L. decemlineata*. These results are somewhat similar to those reported by Lin et al. 1993. Although crop rotation is not a common practice used in potato growing fields of East Azarbaijan and Ardabil provinces, some crop rotation is done using wheat and alfalfa in rotation with potatoes. Since the size of agricultural fields in the region is usually small (0.5–1 ha) it does not seem this fairly uncommon practice would considerably hinder development of *L. decemlineata* resistance to insecticides. Among the agricultural crops grown in the region, none is considered as an alternate host for *L. decemlineata*, which means the host difference as well as the difference in the type of insecticide used for different hosts would not affect development of resistance in this insect. The history of endosulfan usage and potato production practices in the regions monitored may explain the variation in the observed resistance levels.

## Bostanabaad

The pest found its way to the region in 1992. Agria which is a medium to late maturing cultivar is dominantly planted in Bostanabaad and harbors *L. decemlineata* for over 80 days. The potential for long term damage requires sprays up to four times a year of which two to three of the sprays include endosulfan. Endosulfan has been the major chemical for *L. decemlineata* control since 1997 in this region. The use of this chemical reached its peak in 2003, but has declined in the past three years due to lack of acceptable control. High selection pressure could have been the main cause of the high level of resistance for endosulfan found in Bostanabaad. The high frequency of insecticide application as an important cause of resistance development has also been reported by others (Zhao et al. 2000). Development of resistance in such a fairly short time is not unexpected. Rapid development of *L. decemlineata* resistance to organochlorine (Harris and Svec 1981) and some other insecticides such as carbofuran (Ioannidis et al. 1992) and abamectin (Argentine and Clark 1990) have been reported. Although in 2004 the use of endosulfan was somewhat diminished, the considerable decrease in resistance to endosulfan in 2004 could not be justified solely by the decrease in usage. Instead, it is possible that variation between fields in insect response to the insecticide could have been the major factor involved in this regard. This phenomenon has also been demonstrated in other investigations (Heim et al. 1990; Tissler and Zehnder 1990).

## Ardabil

In Ardabil, chemical control of *L. decemlineata* had relied on phosalone and to a less extent, endosulfan, after its initial outbreak. The use of endosulfan became the major management approach after a considerable decrease in phosalone efficiency around 1992. Heavy spraying with endosulfan for several years in a row was followed by control failures that resulted in abandonment of this chemical in Ardabil after 1997. The main chemicals used for controlling this pest after the decline in efficacy of endosulfan have been organophosphorus, carbamates and nicotinoid insecticides which would eliminate the anticipation of cross resistance to endosulfan. Lower level of resistance in Ardabil compared to Bostanabaad population, is likely due to reversion of a high level of resistance that had led to control failure in 1995–1996. Such a decrease in resistance levels has been reported for DDT-resistant populations of *L. decemlineata* (McDonald 1976). Reduction of resistance level in absence of the pesticide previously used is the basis for resistance management (ffrench-Constant et al. 1993a). Scott et al. 2000 stated that resistant populations of the Australian sheep blowfly, *Lucilia cuprina* lost their resistance to dieldrin in absence of exposure, possibly due to the fitness cost involved. Also, resistance to permethrin (Follett et al. 1993), azinphosmethyl (Zhu et al. 1996) and *Bacillus thuringiensis* Cry3A toxin (Whalon et al. 1993; Alyokhin and Ferro 1999) was associated with fitness cost in resistant strains of *L. decemlineata*. Considering these findings, reduced resistance in the Ardabil population could have been related to a fitness cost involved. The other possibilities for partial justification of this reduction in resistance could have been the cessation of endosulfan application and selection pressure in the area.

## Ajabshir

*L. decemlineata* was observed in potato fields of Ajabshir for the first time in 1989, but its damage was negligible and no regular chemical control was required until 1997. In this region short season potato cultivars are planted and the *L. decemlineata* adults emerge around early May. Because potato plants are harvested in late July, only the first generation requires chemical control and the second generation does not cause major damage because the approaching harvest time usually prevents completion of larval development. Hence, exposure of only a small proportion of the population to insecticides may result in a low frequency of selection toward resistance. The use of insecticides belonging to various groups of chemicals in different fields and years also makes selection pressure for resistance less of a problem in this region. This is in agreement with Heim et al. 1990 who stated that the presence of 3 and 4 generations of *L. decemlineata* with no spraying was one of the main reasons for the delay in development, or low levels of resistance to insecticides. The difference in the number of imidacloprid applications (2–4 times per year) in Long Island populations of *L. decemlineata* has also been suggested as the cause of higher level of resistance to this insecticide compared with a Michigan population with imidacloprid application of once a year (Zhao et al. 2000). Although the availability of the host plant for a limited time and consequently reduction in selection pressure can delay development of resistance due to the high capability of *L. decemlineata* to adapt to local climatic and potato growing conditions (Senanayake et al. 2000), regular monitoring of the resistance status in this population must also be taken into consideration. Fipronil is a newly registered insecticide in Iran. Since in some cyclodiene-resistant insects cross resistance to fipronil has been found (Bloomquist 2001; Kristensen et al. 2005), considering fipronil for use in *L. decemlineata* management programs should be done with a thorough knowledge of the resistance status of this pest to cyclodiene insecticides.

## Conclusion

As the high level of endosulfan resistance in the Bostanabaad strain could not be justified by synergism and biochemical studies, further investigation should be done. Target site insensitivity has been reported as the major cause of resistance to cyclodienes (ffrench-Constant et al. 1993b; Anthony et al. 1995; Bass et al. 2004). Additional experiments are required to further investigate mechanisms involved in the endosulfan resistant strain of *L. decemlineata*.

## Abbreviations

CDNB: 1-chloro-2, 4-dinitrobenzene
DEF: S,S,S-tributylphosphorotrithioate
